# Evaluating Human Papillomavirus Vaccination Programs

**DOI:** 10.3201/eid1011.040222

**Published:** 2004-11

**Authors:** Al V. Taira, Christopher P. Neukermans, Gillian D. Sanders

**Affiliations:** * Stanford School of Medicine, Stanford, California, USA;; † Stanford University, Stanford, California, USA;; ‡ Duke University, Durham, North Carolina, USA

**Keywords:** Human papillomavirus, Vaccines, Cost-benefit analysis, Cervix neoplasms, Public health

## Abstract

Human papillomavirus (HPV) has been implicated as the primary etiologic agent of cervical cancer. Potential vaccines against high-risk HPV types are in clinical trials. We evaluated vaccination programs with a vaccine against HPV-16 and HPV-18. We developed disease transmission models that estimated HPV prevalence and infection rates for the population overall, by age group, by level of sexual activity within each age group, and by sex. Data were based on clinical trials and published and unpublished sources. An HPV-16/18 vaccine for 12-year-old girls would reduce cohort cervical cancer cases by 61.8%, with a cost-effectiveness ratio of $14,583 per quality-adjusted life year (QALY). Including male participants in a vaccine rollout would further reduce cervical cancer cases by 2.2% at an incremental cost-effectiveness ratio of $442,039/QALY compared to female-only vaccination. Vaccination against HPV-16 and HPV-18 can be cost-effective, although including male participants in a vaccination program is generally not cost-effective, compared to female-only vaccination.

With 370,000 cases per year and a death rate of approximately 50%, cervical cancer is the third most common malignancy in women worldwide (*1**,**2*). Epidemiologic and laboratory evidence has implicated certain types of human papillomavirus (HPV) as the etiologic agents of cervical cancer (*3**,**4*). On the basis of this evidence, effort is under way to develop an HPV vaccine that targets these oncogenic HPV types (*5*).

Clinical trials of preliminary vaccines in humans began in the late 1990s (*6*). Recent data from an ongoing phase II trial (*7*) look very positive, demonstrating that an HPV-16 vaccine can prevent HPV infection and precancerous lesions in vaccinated women. These data provide hope that an HPV vaccine may be a reality within 5 to 10 years. Public health officials will then need to make important decisions regarding who and when to vaccinate and what level of vaccine penetration is necessary to substantially reduce disease prevalence.

Central to this discussion is the question of whether both sexes should be vaccinated. The general assumption in the literature is that men and boys should be vaccinated (*5**,**6**,**8**,**9*). Although long-term sequelae of HPV infection for men is on average less serious (particularly for heterosexual men), men act as vectors for infection. Including men and boys in a vaccine program would enhance herd immunity and decrease overall incidence of cervical cancer. In this article, we evaluate the benefit and cost-effectiveness of adopting a vaccination strategy for both sexes, compared with that of adopting a female-only strategy. The incremental cost-effectiveness of a vaccination rollout strategy is calculated by dividing the difference in costs between strategies by the difference in quality-adjusted life expectancy.

Because results of the long-term phase III/IV trial are not available, the efficacy of the HPV vaccine is still unknown. Also, acceptance of an HPV vaccine is likely to vary substantially. Resistance to a vaccine may arise because HPV is a sexually transmitted disease (*6**,**10*), although recent studies suggest that an HPV vaccine may be reasonably well accepted (*11*). We therefore evaluated a wide range of vaccine efficacies and population penetrations to understand what is required for a female-only program to achieve sizeable benefit and to identify the scenarios in which incremental male vaccination makes most sense.

## Methods

To capture the effect of a male vaccination program on female HPV infection rates and cervical cancer incidence, we needed to directly model the effect of vaccination on HPV disease transmission dynamics. Therefore, we developed disease-transmission models for HPV-16 and HPV-18, the types associated with most cervical cancer cases and the most likely to be included in HPV vaccines (*3**,**6*). For both types, the transmission models estimated HPV prevalence and infection rates for the U.S. population overall, by age group, level of sexual activity, and sex. The models also enabled us to evaluate the effect of various vaccination programs on prevalence and infection rates.

Long-term equilibrium infection rates by age group, by level of sexual activity, and by sex for each vaccination scenario were determined in the transmission model. These infection rates were then incorporated into a probabilistic decision model. This model estimated the annual incidence of HPV-related precancerous lesions, lifetime cases of invasive cervical cancer, resulting cervical cancer deaths, and total cost of care for a given set of age-specific infection rates. By using the combination of the transmission and decision model, we estimated the effectiveness and cost-effectiveness of alternative vaccine rollout strategies.

### Transmission Model Structure

We used Stella software (v7.0.3, High Performance Systems, Hanover, NH) to develop deterministic transmission models for heterosexual transmission of HPV types 16 and 18. Because level of sexual activity and HPV prevalence are highly age-dependent, we divided the population into nine age categories, from age 12 to age 50. We further divided each age category into four subcategories based on level of sexual activity (Table 1). HPV prevalence among their pool of sex partners, infectivity per infected partner, HPV shedding duration, and HPV infection rates were estimated for each age and activity group to develop a natural history transmission model. Vaccine penetration and efficacy were added to evaluate the effect of potential vaccine programs.

**Table 1:** Input variables^a,b^

Age category (y)	New sex partners/y (%) (12–14)								Mixing between age categories (%) (12–14)^c^						Initial HPV prevalence (%) (15–18)^d^				Duration HPV shedding (%) (19–21)^e^	
	Female				Male				Female			Male			HPV 16		HPV 18			
	0	1	2–4	5+	0	1	2–4	5+	<	=	>	<	=	>	Female	Male	Female	Male	Stop shedding	Completely regress
<18	64	30	5	1	57	30	11	1		64	36		90	10	2.6	3.5	0.9	1.2	55	49
18–20	55	26	15	4	50	25	19	6	1	61	38	18	72	11	4.3	5.0	1.8	2.1	55	49
21–23	55	26	15	4	50	25	19	6	3	59	38	32	58	10	4.6	5.0	2.2	2.3	55	49
24–26	76	19	4	1	66	21	12	2	11	51	38	34	56	10	3.0	3.4	1.5	1.7	37	33
27–29	83	12	4	1	71	15	12	2	11	51	38	34	56	10	1.7	2.7	0.8	1.4	37	33
30–34	89	7	4	1	76	10	12	2	10	49	41	38	49	13	1.0	2.1	0.5	1.1	37	7
35–39	90	6	3	0	81	9	9	1	13	50	37	36	49	15	0.7	1.5	0.4	0.8	37	7
40–44	90	6	3	0	81	9	9	1	15	48	37	39	47	15	0.5	1.1	0.3	0.6	37	7
>45	94	5	1	0	90	6	3	0	15	85		39	62		0.4	0.7	0.2	0.4	37	7

^a^HPV, human papillomavirus. ^b^Mixing between sexual activity categories was assumed to be assortive (22). Relative preference for within-group mixing was estimated by [% of potential partners in group X] / [(% of potential partners in group X) + α(1 – % of potential partners in group X)], where α (the assortment variable) ranged from 0.4 for relatively assortive mixing (base-case value) to –0.4 for relatively disassortive mixing. ^c^For each age group, percentage of partners for persons who are in younger (<), the same (=), or older (>) age groups. ^d^Estimate of the prevaccination natural history of HPV infection. ^e^Probability that within 1 year a person of a given age group will stop shedding HPV and the probability that HPV infection will completely regress.

**Figure 1:**
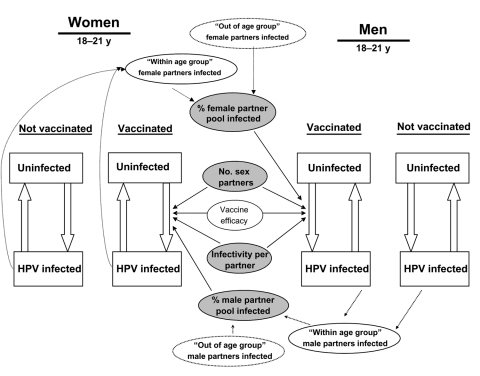
Schematic of the transmission model. The model is divided into nine age categories, with four subcategories per age group (not shown) based on different levels of sexual activity. In each period, uninfected persons can become infected. Infection rates are based on number of sexual partners per year, infectivity per infected partner, and percentage of potential partners who are infected. These variables are age- and risk-group specific. Infection rates for vaccinated persons also depend on the estimated vaccine efficacy. Percentage of potential partners infected includes partners within an age group and potential partners from younger and older age groups. Estimated mixing patterns between age groups differ by sex and age category.

In our analysis, persons of both sexes were either HPV infected or uninfected at the beginning of each time period. In each period, uninfected persons could remain uninfected or become infected, on the basis of infection rates by age category (Figure 1). Infection rates were determined by number of sex partners, HPV prevalence among pool of sex partners, and infectivity per infected partner. HPV prevalence among the pool of sex partners was a function of HPV prevalence by age and risk group, and by sexual mixing patterns (preference of partners in different age groups for partners in different sexual classes) between age groups and between high- and low-risk sexual activity groups (Table 1). Details regarding the transmission model can be found in the Appendix.

### Transmission Model Data

#### Sex Partnering

The level of sexual activity and mixing patterns between subgroups can affect the transmission dynamics of a sexually transmitted disease (*23**,**24*). Table 1 shows our estimates for these variables, based on a survey of the published literature. On average, the number of new sex partners per year for a person in our cohort increases from onset of sexual activity to age 24 and then decreases through age 50 (*12**-**14*). Mixing between sexual activity groups was assumed to be assortive, with a moderate preference to select partners in similar sexual activity groups (*22*). Mixing between age groups was predominantly older men with younger women (*12**-**14*).

#### Duration of HPV Shedding

Persons infected with HPV in a given period are assumed initially to be actively shedding virus and therefore contagious. In subsequent periods, infections can completely resolve or become dormant. Persons whose infections resolve before precancerous lesions develop are assumed to be at no risk for HPV-related cervical cancers, unless they become reinfected with the virus. Persons for whom the virus has gone into a dormant state can no longer transmit the virus, but they remain at increased risk for precancerous lesions and cancer in the future (Table 1).

#### Infectivity per Infected Partner

By using our estimates of HPV prevalence among pools of sex partners, numbers of new sex partners, sexual mixing patterns, and duration of HPV shedding, we derived estimates for infectivity per infected partner for persons of both sexes in each age group in the absence of a vaccination program. Infectivity was highest for women and men <18 years, at ≈0.35 infections per infected partner. This number dropped gradually for older age categories (to ≈0.15 infections per infected partner), representing increased resistance to infection and possible changes in sexual activity and practices in these age groups.

#### HPV Vaccine Characteristics

We assumed that the HPV vaccine would initially be administered by a series of three injections to 12-year-old girls. In our base-case analysis, booster shots would be required for persons in their early 20s. In this scenario, the protective effect of the vaccine lasts for 10 years after the most recent booster. We assumed that the vaccine had 90% efficacy against both HPV-16 and HPV-18 and was given to girls at age 12, with a booster at 22. We assumed 70% of girls were vaccinated, with a vaccine cost of $300 for the initial vaccination (three doses) and $100 for the booster.

### Decision Model Structure and Assumptions

In a previous analysis (*25*), we modeled the overall progression of high-risk oncogenic HPV types to different stages of cervical dysplasia and cancer. In our current analysis, we adapted this model to evaluate the natural history and vaccination scenarios regarding HPV-16 and HPV-18. Estimates regarding Pap screening, lesion treatment, cancer progression and survival, costs, and utilities are based upon our previous analysis (*25*). Specific progression rate of HPV-16 and HPV-18 to different stages of cervical dysplasia and cancer were estimated from the literature (*15**,**19**,**20**,**26**,**27*).

### Model Validation

To validate the model, we compared the incidence of cervical cancer cases and deaths predicted by the prevaccination natural history arm of our model with those reported in the Surveillance, Epidemiology, and End Results (SEER) registry (*28*). Our model’s annual rates of cervical cancer cases and deaths matched 2001 SEER estimates within 10%. The predicted age-specific prevalence of HPV infection in our natural history arm also has a shape and peak of similar magnitude to that reported in the literature (*15**–**18*).

## Results

### Base-Case Analysis

Under our base-case scenario, vaccinated girls would experience a 61.8% overall reduction in acquiring cervical cancers over a lifetime. The analysis predicted, given the current U.S. population of 12-year-old girls (approximately 2.0 million), that the number of expected lifetime cases of cervical cancer related to HPV-16 or HPV-18 would drop from 9,147 to 422, a 95.4% reduction. This strategy would add an average of 6.1 quality-adjusted days of life per woman and have a cost-effectiveness ratio of $14,583 per quality-adjusted life-year (QALY) gained compared to the current environment (Table 2).

**Table 2:** Total discounted healthcare costs, total discounted life expectancy in years, and total quality-adjusted discounted lifetime expectancy in years are presented for prevaccination, and for female-only and male + female vaccination scenarios.

Outcome	No vaccination	HPV-16/18 vaccination	
		Female-only^a^	Female + male^b^
Cost, $	40,423	40,667	40,929
Incremental cost, $		244	261
Life expectancy, y	28.7975	28.8112	28.8117
Incremental life expectancy, d		5.0	0.18
Quality-adjusted life expectancy, y	27.7422	27.7590	27.7596
Incremental quality-adjusted life expectancy, d		6.1	0.21
Incremental cost-effectiveness			
$ per life-year		17,802	534,317
$ per quality-adjusted life-year		14,583	442,039
% reduction in lifetime cervical cancer cases		61.8	2.2

^a^Incremental to no vaccination strategy. ^b^Incremental to a female-only vaccination strategy.

### Vaccinating Men and Boys

If both sexes were vaccinated with an HPV-16/18 vaccine, total cervical cancer cases in that cohort would drop by 63.9%, compared to the number of cases in the scenario before vaccination. The number of cancer cases related to HPV-16 or HPV-18 would decrease from a prevaccination 9,147 to 113, a 98.8% drop from the number in the prevaccination scenario. Expanding the vaccination program to men and boys would add an incremental 0.21 quality-adjusted days of life per woman at a cost-effectiveness ratio of $442,039/QALY compared to the female-only strategy (Table 2).

### Vaccine Penetration and Efficacy

**Figure 2:**
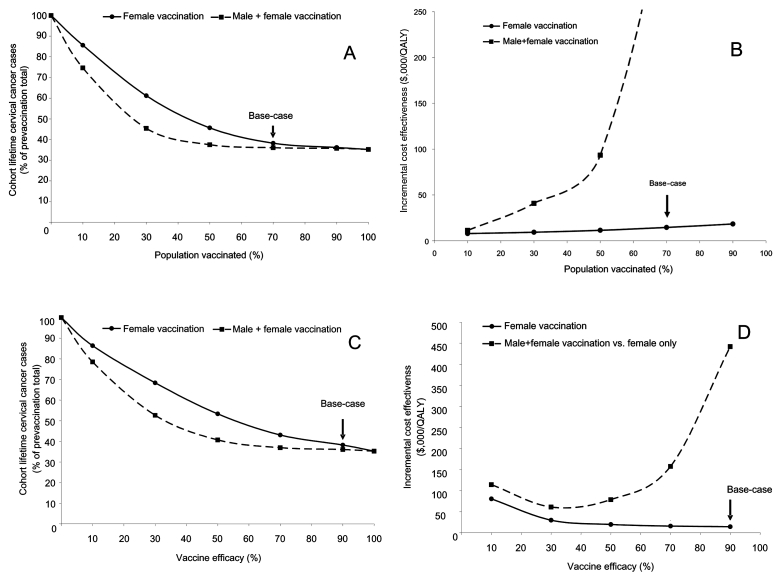
A) Vaccine penetration scenario. Relationship between percentage of the population receiving the vaccine and the number of lifetime cervical cancer cases. The solid line represents a female-only vaccination strategy. The dashed line represents a strategy of vaccinating both sexes. The arrow indicates the base-case scenario of a female-only strategy with 70% penetration. B) Vaccine penetration scenario. Relationship between percentage of the population receiving the vaccine and program cost-effectiveness. The solid line represents the cost-effectiveness ($/quality-adjusted life-year [QALY]) of a female-only vaccination program compared to current practice. The dashed line represents the incremental cost-effectiveness of including male participants in a vaccine program compared to a female-only strategy. The arrow indicates the base-case scenario of a female-only program with 70% penetration. C) Vaccine efficacy scenario. Relationship between vaccine efficacy and the number of cohort lifetime cervical cancer cases. The solid line represents a female-only vaccination strategy. The dashed line represents a strategy of vaccinating both sexes. The arrow indicates the base-case scenario of a female-only strategy assuming 90% vaccine efficacy. D) Vaccine efficacy scenario. Relationship between vaccine efficacy and program cost-effectiveness. The solid line represents the cost-effectiveness ($/quality-adjusted life-year [QALY]) of a female-only vaccination program compared to current practice. The dashed line represents the incremental cost-effectiveness of including male participants in a vaccine program compared to a female-only strategy. The arrow indicates the base-case scenario of a female-only program at 90% vaccine efficacy.

Figure 2A shows how varying the vaccine coverage of a female-only HPV-16/18 vaccination program affects the number of lifetime cervical cancer cases. As expected, as vaccine coverage increases, the number of cervical cancer cases decreases. However, based on scenarios that used our transmission model, the relationship is not linear. Because of the benefits of herd immunity, vaccinating even a relatively small portion of the target population leads to substantial decreases in disease prevalence and resulting negative sequelae relative to prevaccination rates. Figure 2A also illustrates the effect of vaccinating both sexes. A combined male-female program always results in lower levels of cohort cervical cancer cases than a female-only program. However, this difference is only large when levels of female vaccine penetration are low.

Figure 2B shows the cost-effectiveness of HPV-16/18 vaccination programs compared to the current environment as coverage varies. The cost-effectiveness of female-only vaccination is attractive at all ranges of vaccine penetration. At lower vaccine penetration levels, including male participants in the vaccination program also becomes cost-effective. For example, at 30% female vaccine penetration, including male participants is reasonably cost-effective at $40,865/QALY compared to vaccinating female participants only. Figure 2C and D show similar data for changes in vaccine efficacy.

### Vaccination Age

Our analysis assumes that vaccination would focus on children 12 years of age. We considered alternative vaccination strategies that would focus on either infants or persons 18 years of age. Because most women are not sexually active until after age 12, focusing on infants or 12-year-old children leads to approximately the same decrease in lifetime cases of cervical cancer. However, delaying initial vaccination until age 18 leads to only a 54.7% decrease in the number of cancer cases in this cohort. If focusing on the older age group also leads to a decrease in vaccine penetration (60%), then program effectiveness drops further to a 50.9% decrease in lifetime cervical cancer cases in this cohort.

We also considered how the optimal vaccination age was affected if the efficacy of the vaccine waned. If the vaccine efficacy waned over 10 years and no booster was provided, a vaccination program that targeted 18-year-old women would dominate one which targeted 12-year-old girls. In this scenario the cost-effectiveness of also vaccinating 18-year-old men would be economically favorable, with a cost-effectiveness of $57,795/QALY compared to the cost-effectiveness of vaccinating women only. If, however, two booster shots were given at 5-year intervals to maintain the vaccine's efficacy, 12-year-old girls would return to being the optimal vaccination group, but the cost-effectiveness of vaccinating boys would increase to $388,368/QALY.

### Effect of Vaccination over Time and Catch-up Vaccination

Under our base-case scenario with an HPV-16/18 vaccine, the first cohort of vaccinated 12-year-old girls would experience a 29.7% decrease in overall cervical cancer cases at a cost-effectiveness of $27,566/QALY, compared to their experience without vaccination. Vaccinating boys would cost $285,776/QALY compared with a female-only program to reduce cervical cancer cases an additional 4.7%. In time, however, lifetime cervical cancer cases would reach a steady-state of 62% of prevaccination level. Thus, even the first cohort would experience almost half of the achievable benefit of a long-term vaccination program. Table 3 displays the average reduction in lifetime cervical cancer risk for girls vaccinated at age 12 through a large-scale vaccination program, compared to the reduction in risk to women ages 24 and 30 who opt for catch-up vaccination once a vaccine becomes available.

**Table 3:** Reduction in lifetime risk of cervical cancer

Cohort	% reduction in lifetime risk of cervical cancer
Full potential of program in 12-year-old girls	64
First cohort of 12-year-old girls vaccinated^a^	46
24-year-old women who receive catch-up vaccination^b^	35
30-year-old women who receive catch-up vaccination^b^	17

^a^This group experiences a lower reduction in cancer cases because many of their sex partners will be drawn from a population pool that has not been vaccinated. ^b^24- or 30-year-old women who opt for catch-up vaccination in the first year that the vaccine becomes available.

### Pap Screening Guidelines

**Figure 3:**
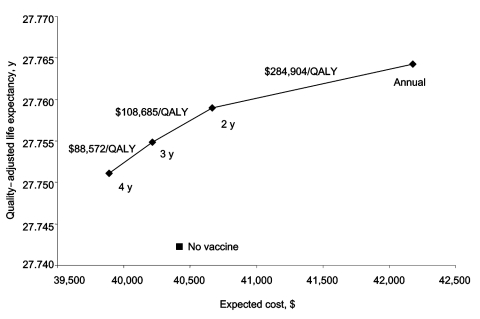
Effect of changing frequency with which vaccinated women receive a Pap test. The diamonds represent Pap testing annually, every 2 years (base case), every 3 years, and every 4 years. The x-axis represents the lifetime expected cost of the vaccination strategy; the y-axis is the quality-adjusted life expectancy in years. The incremental cost-effectiveness of increasing the frequency of Pap testing for vaccinated women is indicated numerically above the cost-effectiveness frontier. QALY, quality-adjusted life-year.

Although an HPV-16/18 vaccine would not protect against all oncogenic HPV strains, we wanted to explore whether the vaccine could sufficiently reduce the prevalence of cervical cancer and precancerous lesions to allow for less frequent cervical cancer screening. Our base-case analysis assumes that 71% of women get Pap smears every 2 years (*29*). Figure 3 presents the cost-effectiveness of moving to more or less frequent screening intervals, in the presence of an established vaccine program.

### Sensitivity Analyses

**Figure 4:**
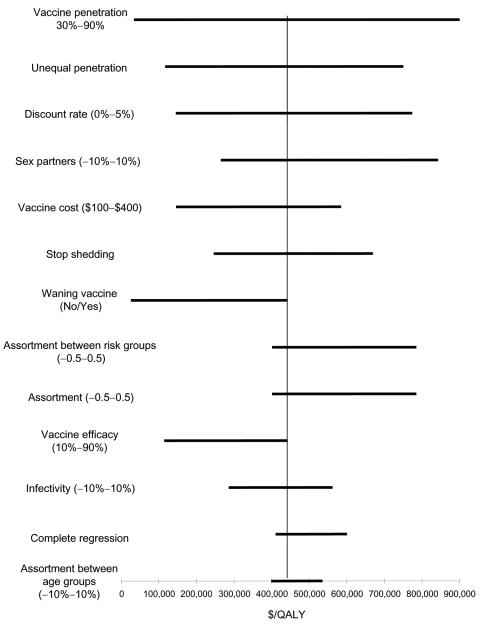
Tornado diagram representing the incremental cost-effectiveness ratios of one-way sensitivity analysis on vaccinating men and women compared to vaccinating women only. The vertical line represents the incremental cost-effectiveness ratio under base-case conditions. The sensitivity analysis range is displayed in parentheses next to each variable. Unequal penetration represents potential for lower (or higher) vaccine penetration in the highest risk groups, from 30% to 80% of target group, compared to 70% penetration in base case. QALY, quality-adjusted life-year.

We performed sensitivity analyses on a range of model variables. The female-only vaccination program remained economically attractive under a wide range of variable assumptions. However, the incremental benefit of vaccinating men and boys was sensitive to changes in key variables. Figure 4 shows one-way sensitivity analyses of the cost-effectiveness of incrementally vaccinating male participants compared to the cost-effectiveness of female-only vaccination.

## Discussion

By using a disease transmission model for the sexual transmission of HPV, we demonstrated that an HPV-16/18 vaccine would be cost-effective and could reduce lifetime cervical cancer cases by 61.8%. Although a universal vaccination program would have the greatest benefit, because of the benefits of herd immunity, a program that achieves even 70% coverage would dramatically reduce cohort lifetime cervical cancer cases.

Although the literature often suggests that men and boys should be included in an HPV vaccination program (*5**,**6**,**8**,**9*), our results suggest that this strategy may not be the most cost-effective public health strategy. Under our base-case assumptions, including men and boys in a vaccination program would further reduce infections and cancer cases only slightly, with an unattractive cost-effectiveness ratio of $442,039/QALY saved. In addition, the absolute cost of expanding coverage to men and boys is high. Assuming a $300 vaccine, achieving 50%-70% coverage for the current U.S. population of approximately 2.1 million 12-year-old boys would cost >$300 million annually.

In certain scenarios, such as those in which vaccine efficacy wanes rapidly without boosters or overall vaccine coverage is low, vaccinating male participants can have a substantial effect (Figure 4). In a recent article that modeled risk groups but not age groups, Hughes et al. (*30*) found that for a single-type HPV vaccine with a 10-year mean duration and no booster that was meant for 16-year-olds, a program focusing on girls would have only two thirds of the impact on HPV infection rates as a program focusing on both sexes. Modeling both risk and age groups, we found that the incremental cost-effectiveness ratio of vaccinating boys dropped to $51,646/QALY for a vaccine with rapidly waning efficacy and no booster. Also, if vaccination rates are lower among the most sexually active girls, the female-only vaccination strategy will be less effective. In sensitivity analyses, we demonstrated that vaccinating boys in such a situation would be reasonably cost-effective. For example, if vaccine penetration amongst the highest risk girls reached only 30%, the cost-effectiveness ratio of vaccinating boys drops from $442,039/QALY to $116,413/QALY. Nonetheless, even in this scenario, vaccinating boys is less cost-effective than achieving higher vaccine penetration in girls at high risk (analysis not shown).

We demonstrated that vaccinating women at the onset of sexual activity is cost-effective and will lead to the greatest reduction in cervical cancer incidence. Because we assume that the vaccine will require a booster after 10 years, focusing on 12-year-olds would be more cost-effective than focusing on infants ($27,600/QALY). If a vaccination program focusing on infants were more widely accepted, with initial coverage of 80% versus 70% in the base-case scenario, we would expect only an additional 1.2% decrease in overall lifetime incidence of cervical cancer, and the cost-effectiveness ratio would increase to $28,181/QALY. Focusing on 18-year-olds would limit the efficacy of the vaccine program and is not recommended unless focusing on younger groups is not possible.

We explored the effect of changing cervical cancer screening interval guidelines once a vaccine program was established (Figure 3). Even in a prevaccination environment, researchers found that moving from screening every 2 years to every year is not particularly cost-effective (*31*). Kulasingam and Myers recently found that Pap testing may be delayed to a later age than currently recommended when an HPV vaccine has been given; although that analysis did not include disease-transmission dynamics and predicted that broad-based immunization would decrease cervical cancer incidence by 17% (*32*). By using a disease-transmission model that predicts greater vaccine impact, we demonstrated that Pap testing vaccinated women every 3 or 4 years had a more powerful effect than a no-vaccine strategy (i.e., cost less and increased quality-adjusted life expectancy). With a vaccine program in place, moving from screening every 3 years to every 2 years cost >$100,000/QALY, while annual screening is not economically favorable (Figure 3). Given these data, with a vaccine program in place, physicians may be comfortable moving to less frequent screening.

We did not include in our analysis the effect of an HPV vaccine on several other cancers associated with HPV. We also did not examine the effect of vaccines targeting the nononcogenic HPV types most commonly associated with genital warts. Including the former would make the vaccine strategies appear to be even more cost-effective. The latter can be considered as a separate analysis, since a vaccine would offer little cross-protection between HPV types (*5*). Also, although some have suggested that lesion treatment protects against sequelae of future HPV infections (e.g., squamous intraepithelial lesions and cervical cancer) (*30*), we are not aware of evidence that supports this hypothesis, so we did not include it in our analysis. Including this potential benefit would diminish the cost-effectiveness of a future vaccine. Finally, our analysis does not examine targeted vaccination in men who are at high risk, for instance, in the community of men who have sex with men, in which HPV infection rates are higher than for the general population.

Although this analysis modeled vaccine programs in the United States, our results may have relevance for decision makers in less developed countries where public health resources are limited and cervical cancer death rates can be markedly higher than in the United States. These countries may have difficulty achieving high levels of vaccine penetration. However, because even modest vaccine coverage appears to substantially reduce cervical cancer cases, a partial vaccination program that includes specific populations might be more efficacious and cost-effective for these countries than alternative options, such as Pap or HPV screening.

Our analysis indicates that vaccinating 12-year-old girls with an HPV-16/18 vaccine would cost $14,583 /QALY, whereas vaccinating boys costs $442,039/QALY. In comparison, screening strategies of women for cervical cancer with Pap smears has been estimated to cost between $7,777 per life-year (LY) (quadrennial screening) and $166,000/LY (annual screening) and depends on the type of testing and prevalence of disease (*31*). Similarly, studies of hepatitis B vaccines have estimated costs from $4,800 to $16,000/QALY to selectively vaccinate at-risk populations versus universal infant vaccination or versus no vaccination, respectively (*33*).

Vaccine evaluations that do not include disease transmission can underestimate actual vaccine benefit (*34**–**36*). By modeling disease transmission by age category and risk grouping, we were able to estimate the effect of herd immunity, which we know from actual vaccine rollouts can be substantial (*37**,**38*). Prior cost-effectiveness analyses of potential HPV vaccines by our group (*25*) and others (*32**,**39*) have not included transmission by age category, multiple sexual activity subgroups, or the protective benefit of herd immunity. As a result, these analyses have likely underestimated the benefits of vaccination. In addition, previous approaches did not attempt to evaluate the cost-effectiveness of male vaccination. By modeling transmission by different age and risk groups, we also were able to address the issue of unequal vaccine penetration in high-risk groups, an important real world phenomenon.

Because an HPV vaccine is likely to be available in the future, public health officials will need to decide on HPV vaccine rollout strategies. Our analysis shows that a vaccine that protects against HPV-16/18 could be cost-effective and has the potential to substantially reduce cervical cancer rates. Additionally, under most scenarios, we showed that including men and boys in a vaccination program has a limited effect, which suggests that scarce healthcare resources could be used in a more productive manner. As ongoing clinical trials and vaccine development progress, we believe our analysis will provide public health officials with the tools needed to make optimal recommendations with limited resources.

## Appendix

To capture the effect of a male vaccination program on female human papillomavirus (HPV) infection rates and cervical cancer incidence, we needed to directly model the effect of vaccination on HPV disease-transmission dynamics. We developed disease-transmission models for HPV-16 and HVP-18, the HPV types associated with most cervical cancer cases and the most likely to be included in HPV vaccines. For both types, the transmission models estimated HPV prevalence and infection rates for the U.S. population overall, by age group, by level of sexual activity, and by sex. The models also enabled us to evaluate the effect of various vaccination scenarios on prevalence and infection rates.

For the status quo and for each vaccination scenario, infection rates by age group for vaccinated and unvaccinated women were estimated by the disease transmission models. Infection rates by age group for vaccinated and unvaccinated women were then incorporated into a probabilistic decision model. The decision model estimated the annual incidence of HPV-related precancerous lesions, lifetime cases of invasive cervical cancer, resulting cervical cancer deaths, and total cost of care for a given set of age-specific infection rates. By using the combination of the transmission and decision model, we were able to estimate the effectiveness (increase in life expectancy and reduction in cervical cancer incidence) and cost-effectiveness of alternative vaccine rollout strategies.

### Transmission Model Structure

In our analysis, both sexes are either HPV infected or uninfected at the beginning of each period. In each period, uninfected persons can remain uninfected or become infected, based on infection rates by age category. *G_a_*_,_*_r_*_,_*_t_*_,_*_v_*_=0_ represents unvaccinated women (*v* = 0) of a given age (*a*), of a given sexual activity subgroup (*r*), in time period (*t*) who are not HPV infected. In the next time period, as these women move into the next age category (*a*+1), they can remain uninfected, assuming the status of *G_a_*_+1,_*_r_*_,_*_t_*_+1,_*_v_*_=0_, or they can become infected, assuming the status of infected women of age (*a*+1) and sexual activity subgroup (*r*), *F_a_*_+1,_*_r_*_,_*_t_*_+1,_*_v_*_=0_. The rate of HPV infection is a function of *p_a_*_,_*_r_*, the number of partners per year for age group (*a*) and sexual activity group (*r*); *i_a_*, the infectivity per infected partner for persons of age (*a*); and *Q_a_*_,_*_r_*, the prevalence of HPV infection in the pool of male partners for women of this age and sexual activity subgroup. Overall prevalence of HPV infection in their specific pool of male partners (*Q_a_*_,_*_r_*,), in turn, depends on the preference of women in this age group for male partners in younger (l*_f_*_,_*_a_*), older (*h_f_*_,_*_a_*), and the same (1–l*_f_*_,_*_a_*–*h_f_*_,_*_a_*) age group and the prevalence of HPV infection in these respective male groups. The resulting rate of HPV infection differs by age group and differs for women in different sexual activity subgroup within the different age groups. Rate of HPV regression, *d_a_*, when active infection clears and at which time a woman is no longer infective, is also age dependent.

Women can enter the transmission model already having been vaccinated or can receive the vaccine at older ages, depending on vaccination scenario being evaluated. The rate of HPV infection for vaccinated women, *G_a_*_,_*_r_*_,_*_t_*_,_*_v_*_=1_, is a function of *p_a_*_,_*_r_*, the number of partners per year; *i_a_*, the infectivity per infected partner for persons of age (*a*); *Q_a_*_,_*_r_*, the prevalence of HPV infection in the pool of male partners for women of this age and sexual activity subgroup; and vaccine efficacy (*e*).

*J_a_*_,_*_r_*_,_*_t_*_,_*_v_* represents men of a given age (*a*), of a given sexual activity subgroup (*r*), in time period (*t*), who are not HPV infected. *K_a_*_,_*_r_*_,_*_t_*_,_*_v_* represents men of a given age (*a*), of a given sexual activity subgroup (*r*), in time period (*t*) who are HPV infected. Infection rates for men and the effect of a vaccine are determined in the same way as for women, though the values of variables differ between men and women of similar age groups (e.g., in most age categories, more men than women prefer partners in lower age groups).

### Scenario Evaluation

During each period a new cohort of approximately 2 million male participants and 2 million female participants enters the model. This cohort is divided into vaccinated and unvaccinated persons. Vaccination rates for a given age cohort are 0%–100%, depending on which scenario is being evaluated. Vaccination rates can also vary by sex, and vaccination rates of persons the same age can differ according to sexual activity risk groups. This variation enables us to evaluate scenarios of differing vaccine penetration by risk category.

Vaccine efficacy (*e*) can vary from 0% to 100%. To capture the effect of alternative vaccines with different durations of effectiveness, we adjust the variable (*e*) for different age groups. For instance, to model a vaccination strategy for 12-year-old girls with a 90% effective vaccine that has a 10-year duration, the variable (*e*) would be set equal to 90% for ages 12–21. For ages 22 or older, efficacy can be set to equal 0%. To model a 10-year booster in the above scenario, the variable (*e*) can also be set equal to 90% for ages 22–31, to reflect the protection of the booster. In addition, gradually waning vaccine efficacy can be modeled by gradually decreasing variable (*e*) from 90% to 0% over a given number of age categories

### Transmission Model Output

**Figure A1:**
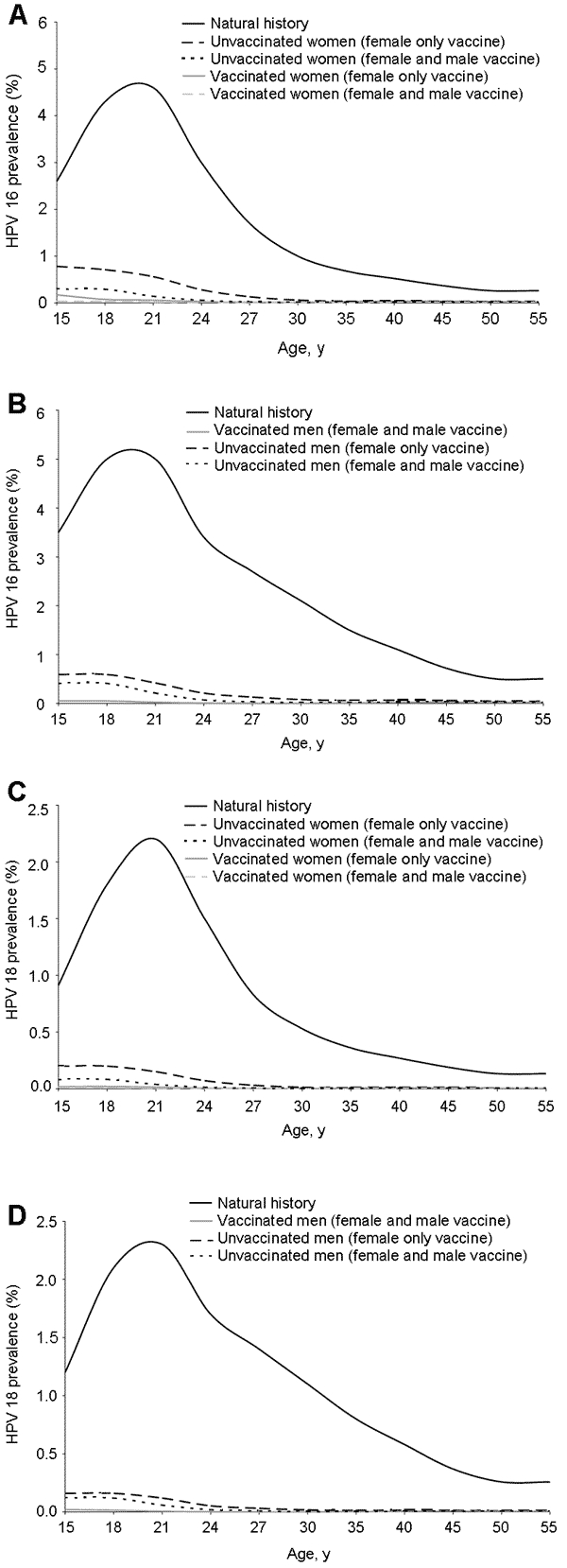
Prevalence of HPV-16 and HPV-18. A) HPV-16 prevalence in women. B) HPV-16 prevalence in men. C) HPV-18 prevalence in women. D) HPV-18 prevalence in men.

Using the transmission model, we estimated the prevalence of HPV-16 and HPV-18 infection by age group, in the absence of a vaccine (Figure A1). For both HPV-16 and HPV-18, prevalence increases rapidly after sexual debut, peaking in women and men in their early 20s. Prevalence drops off for persons in their late 20s and early 30s, which corresponds to a drop in the number of new sex partners per year in these age groups. For both men and women overall (including all sexual activity categories), population prevalence of HPV-16 peaks at ≈5% and population prevalence of HPV-18 peaks at ≈2%, Prevalence decreases to <1% for both HPV types for persons >40 years old. The predicted age-specific prevalence of HPV infection in our natural history arm has a shape and peak of similar magnitude to that seen in the literature (*40**–**43*).

**Figure A2:**
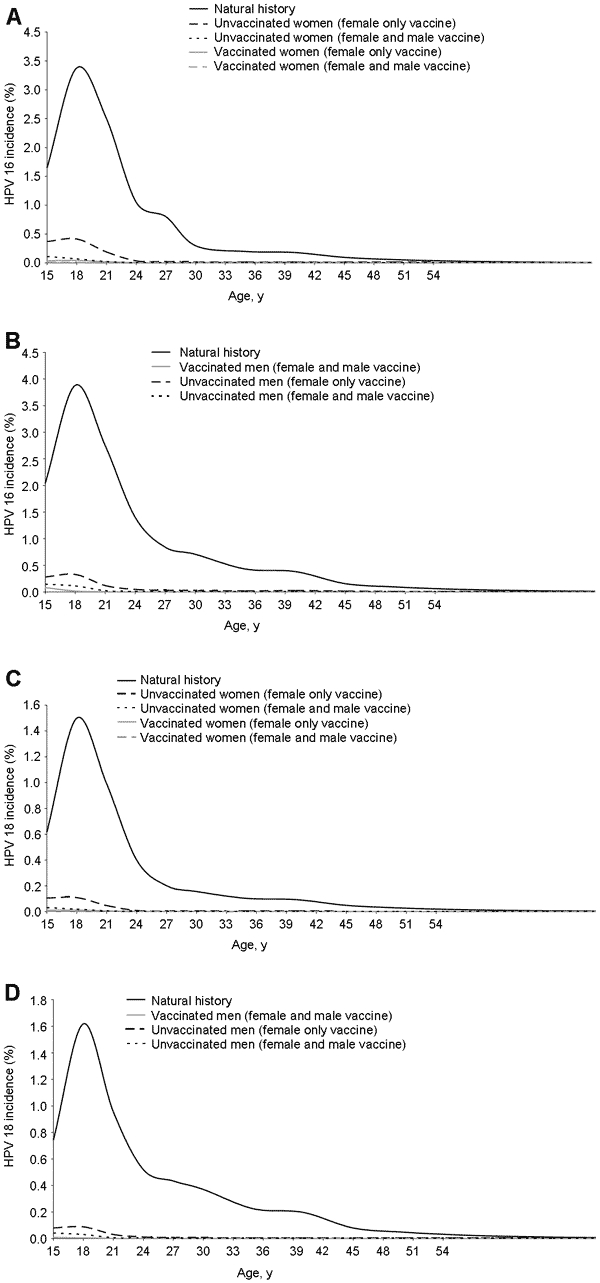
Incidence of HPV-16 and HPV-18. A) HPV-16 incidence in women. B) HPV-16 incidence in men. C) HPV-18 incidence in women. D) HPV-18 incidence in men.

We evaluated various vaccination program scenarios to assess how different vaccination program options would affect HPV transmission and prevalence. We used the transmission model to estimate the incidence and prevalence of HPV-16 and HPV-18 by age group for both vaccinated and unvaccinated women under two main vaccination scenarios. First, we evaluated the effect of vaccinating only female participants. We then compared that to the effect of vaccinating both sexes. Results are presented in Figure A1 and A2.

### Decision Model

In a previous analysis (*44*), we developed a probabilistic Markov decision model to estimate the progression of high-risk oncogenic HPV types to different stages of cervical dysplasia or cancer and to evaluate different female-only vaccine strategies. In our original modeling approach, we assumed that vaccinated women would have reduced HPV infection rates that equaled the estimated vaccine efficacy. Also, in the original model, unvaccinated women had no decrease in HPV infection rates, despite the presence of a widespread vaccination program. In truth, the effect of a vaccine on HPV infection rates for vaccinated women depends on both the vaccine efficacy and the overall impact of the vaccination program at reducing population prevalence of HPV in both men and women. Additionally, a vaccine program will reduce infection rates for unvaccinated women by a herd immunity benefit. Our modeling approach includes these factors.

In our approach, we use the transmission model described above to estimate the reduction in HPV infection rates for both vaccinated and unvaccinated women under a female-only vaccination strategy. For each age group, for vaccinated and unvaccinated women, these infection rates are incorporated into the Markov model, which then estimates disease progression, cervical cancer incidence, cervical cancer deaths and associated costs, and health utilities under a female-only vaccine program. In this way, we capture both the direct vaccine effect and the indirect benefit of herd immunity to both vaccinated and unvaccinated women for a female-only vaccine program.

Next, we used the transmission model to estimate the reduction in HPV infection rates for both vaccinated and unvaccinated women when both sexes are vaccinated. These scenario-specific infection rates for each age group and for both vaccinated and unvaccinated women are included in the Markov model, and the costs and health benefits are evaluated.
